# 1-(2-Chloro­phen­yl)-2-(2-methyl-5-phenyl-3-thien­yl)-3,3,4,4,5,5-hexa­fluoro­cyclo­pent-1-ene: a new photochromic diaryl­ethene

**DOI:** 10.1107/S1600536808013330

**Published:** 2008-05-10

**Authors:** Shanshan Gong, Congbin Fan, Weijun Liu, Gang Liu

**Affiliations:** a Jiangxi Key Laboratory of Organic Chemistry, Jiangxi Science & Technology Normal University, Nanchang 330013, People’s Republic of China

## Abstract

The title compound, C_22_H_13_ClF_6_S, is a hybrid diaryl­ethene derivative with one 3-thienyl substituent, and a Cl-substituted six-membered aryl unit bonded to the double bond of a hexa­fluoro­cyclo­pentene ring. In the crystal structure, the mol­ecule adopts a photo-active anti­parallel conformation that can undergo effective photocyclization reactions. The distance between the two reactive C atoms is 3.848 (3) Å. The dihedral angles between the least-squares cyclo­pentene plane and those of the adjacent thio­phene and chloro­phenyl rings are 49.39 (8) and 59.88 (8)°, respectively. The F atoms are disordered over two positions, with site occupancy factors of 0.6 and 0.4.

## Related literature

For related literature, see: Dürr & Bouas-Laurent (1990 ▶); Irie (2000 ▶); Kobatake & Irie (2004 ▶); Ramamurthy & Venkatesan (1987 ▶); Tian & Yang (2004 ▶); Woodward & Hoffmann (1970 ▶); Zheng *et al.* (2007 ▶); Peters *et al.* (2003 ▶).
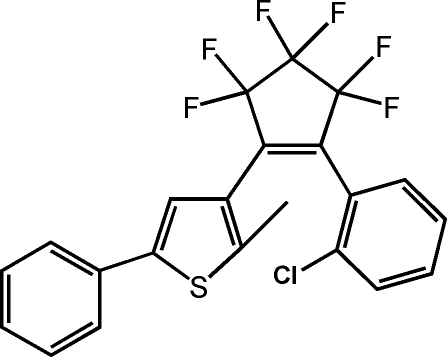


## Experimental

### 

#### Crystal data

C_22_H_13_ClF_6_S
*M* *_r_* = 458.83
Triclinic, 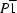

*a* = 8.8064 (10) Å
*b* = 10.4185 (12) Å
*c* = 11.6563 (13) Å
α = 85.265 (1)°
β = 76.935 (1)°
γ = 76.324 (1)°
*V* = 1011.8 (2) Å^3^
*Z* = 2
Mo *K*α radiation
μ = 0.35 mm^−1^
*T* = 291 (2) K
0.46 × 0.37 × 0.27 mm

#### Data collection

Bruker SMART CCD area-detector diffractometer
Absorption correction: multi-scan (*SADABS*; Sheldrick, 1996 ▶) *T*_min_ = 0.835, *T*_max_ = 0.910
7557 measured reflections
3741 independent reflections
3039 reflections with *I* > 2σ(*I*)
*R*_int_ = 0.013

#### Refinement

*R*[*F* ^2^ > 2σ(*F* ^2^)] = 0.036
*wR*(*F* ^2^) = 0.100
*S* = 1.03
3741 reflections
326 parameters
66 restraints
H-atom parameters constrained
Δρ_max_ = 0.15 e Å^−3^
Δρ_min_ = −0.17 e Å^−3^

### 

Data collection: *SMART* (Bruker,1997 ▶); cell refinement: *SAINT* (Bruker, 1997 ▶); data reduction: *SAINT*; program(s) used to solve structure: *SHELXS97* (Sheldrick, 2008 ▶); program(s) used to refine structure: *SHELXL97* (Sheldrick, 2008 ▶); molecular graphics: *ORTEPIII* (Burnett & Johnson, 1996 ▶) and *ORTEP-3 for Windows* (Farrugia, 1997 ▶); software used to prepare material for publication: *SHELXL97*.

## Supplementary Material

Crystal structure: contains datablocks I, global. DOI: 10.1107/S1600536808013330/dn2342sup1.cif

Structure factors: contains datablocks I. DOI: 10.1107/S1600536808013330/dn2342Isup2.hkl

Additional supplementary materials:  crystallographic information; 3D view; checkCIF report

## Figures and Tables

**Fig. 1.**
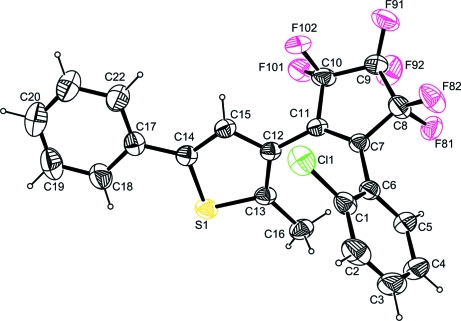
Molecular view of title compound with the atom-labelling scheme. Ellipsoids are drawn at the 30% probability level. H atoms are represented as small spheres of arbitrary radii. Only the major component of the disordered F atoms are shown for clarity.

**Fig. 2.**
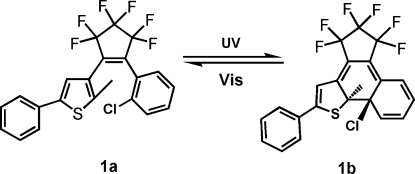
Photochromic interconvertion of the title compound.

**Fig. 3.**
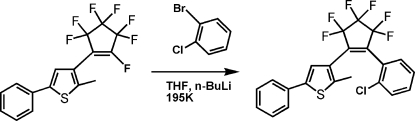
Synthesis of the title compound.

## supplementary crystallographic information

### Comment

Organic photochromic materials have attracted much attention, because of their potential application to optical memory media and optical switches. (Dürr & Bouas-Laurent, 1990; Tian & Yang, 2004). Among all organic photochromic compounds, diarylethenes with heterocyclic aryl groups are the most promising candidates for those application, mainly due to the excellent thermal stability of the respective isomers, notable fatigue resistance, and high reactivity in the solid state (Irie, 2000). The backbone of all photochromic perfluorocyclopentene systems are composed of five-membered heterocyclic rings (Zheng *et al.*, 2007) or the combination of a five-membered aryl ring and a vinyl group. we decided to investigate if replacing the five-membered heterocyclic ring in the diarylethene with a six-membered aryl ring would induce novel characteristics. This paper presents the synthesis and crystal structure of the title compound a six-membered aryl ring group bearing a Cl atom.

The thienyl and the 2-chlorophenyl rings are in cis-position with respect to the C7=C11 double bond (Fig. 1). They are located on each side of the fluorocyclopentene ring, as reflected by the torsion angles C1—C6—C7—C11 [-60.52 ( 0.31)°] and C7—C11—C12—C13 [45.95 (23)°]. The dihedral angles between the least-square cyclopentene plane and those of the adjacent thiophene and chloro-phenyl rings are 49.39 (8)° and 59.88 (8)° respectively.

Such conformation is crucial for the compound to exhibit photochromic and photoinduced properties (Woodward & Hoffmann, 1970). The intramolecular distance between the two reactive C atoms (C1—C13) is 3.848 (3) Å. This distance indicates that the crystal can be expected to undergo photochromism to form compound (Ib)( Fig. 2), because photochromic reactivity usually appears when distance between the reactive C atoms is less than 4.2 Å (Ramamurthy & Venkatesan, 1987; Kobatake *et al.*, 2004). Crystal of (Ib) shows photochromism in accordance with the expected ring closure to form (Ib). Upon irradiation with 313 nm light, the colorless single-crystal of (Ia) turned red quickly. When the red crystal was dissolved in hexane, the solution also showed a red color, with an absorption maximum at 523 nm, consistent with the presence of the closed-ring isomer (Ib). Upon irradiation with visible light with wavelength greater than 510 nm, the red crystal can return to its initial colorless state, and the absorption spectrum of the hexane solution containing the colorless crystal is the same as that of solution of the open-ring form, (Ia), with the absorption maximum at 273 nm.

### Experimental

Compound (Ia) was prepared from (2-methyl-5-phenyl-3-thienyl)- 3,3,4,4,5,5-hexafluorocyclopent-1-ene (1.83 g, 5.00 mmol) (Peters *et al.*, 2003) and 2-bromo-chlorobenzene (0.96 g, 5.00 mmol). To a stirred solution of compound 2-bromo-chlorobenzene (0.96 g, 5.00 mmol) in THF (80 ml) was added dropwise a 2.5 mol/*L* n-BuLi in hexane (2.0 ml) at 195 K under a nitrogen atmosphere (Fig. 3). Stirring was continued for 30 min, (2-methyl-5-phenyl-3-thienyl)- 3,3,4,4,5,5-hexafluorocyclopent-1-ene (1.83 g, 5.00 mmol) was slowly added to the reaction mixture, and the mixture was stirred for 2.0 h at 195 K. The reaction was stopped by the addition of water. Through a series of operations, [1-(2-methyl-5-phenyl-3-thienyl), 2-(2-chlorophenyl)]- 3,3,4,4,5,5-hexafluorocyclopent-1-ene (1.32 g, 2.88 mmol) was obtained in 57.5% yield by column chromatography on SiO_2_ using hexane as the eluent. Finally the colorless crystals were obtained by slow vapour diffusion of chloroform and hexane(1:2). The title compound was characterized by melting point, elemental analysis and NMR(m.p.361.1–361.7 K). ^1^HNMR (400 MHz, CDCl_3_, TMS): δ 2.08 (s, 3H, –CH_3_), 7.14 (s, 1H, thiophene-H), 7.27, 7.29 (d, 2H, *J* = 8.0 Hz, benzene-H), 7.33–7.38 (m, 4H, benzene-H), 7.40 (m, 1H, benzene-H), 7.46, 7.48 (d, 2H, *J* = 8.0 Hz, benzene-H); ^13^C NMR (100 MHz, CDCl_3_): δ 14.55, 123.10, 125.11, 125.59, 126.98, 127.75, 128.91, 130.43, 130.55, 131.25, 133.45, 133.70, 140.70, 141.73; IR (KBr, cm^-1^): 754, 853, 987, 1053, 1132, 1192, 1272, 1330, 1441, 1473, 1502, 1598, 1674, 2925, 3064; Anal. Calcd. for C_24_H_19_ClF_6_S(%): C, 57.59, H, 2.86, Found: C, 57.28, H, 2.59.

### Refinement

All H atoms attached to C were fixed geometrically and treated as riding with C—H = 0.96 Å (methyl) or 0.93 Å (aromatic) with U_iso_(H) = 1.2U_eq_(aromatic) or U_iso_(H) = 1.5U_eq_(methyl).

The F atoms attached to the cyclopentene ring are disordered over two positions. The occupancy factors of the two positions were refined using an overall isotropic thermal parameter and by restraining the sum of the occupancy to remain equal to 1.0. The ratio between the two occupancies was found to be 0.6/0.4. The C-F distances were restrained using SADI (SHELXL-97) instructions and similar Uij restraints as well as rigid bond restraints were used in the final refinement cycles.

### Crystal data

**Table:**

C_22_H_13_ClF_6_S	*Z* = 2
*M**_r_* = 458.83	*F*_000_ = 464
Triclinic, *P*1	*D*_x_ = 1.506 Mg m^−^^3^
Hall symbol: -P 1	Melting point: 361 K
*a* = 8.8064 (10) Å	Mo *K*α radiation λ = 0.71073 Å
*b* = 10.4185 (12) Å	Cell parameters from 3385 reflections
*c* = 11.6563 (13) Å	θ = 2.4–26.1º
α = 85.265 (1)º	µ = 0.35 mm^−^^1^
β = 76.935 (1)º	*T* = 291 (2) K
γ = 76.324 (1)º	Block, colorless
*V* = 1011.8 (2) Å^3^	0.46 × 0.37 × 0.27 mm

### Data collection

**Table:**

Bruker SMART CCD area-detector diffractometer	3741 independent reflections
Radiation source: fine-focus sealed tube	3039 reflections with *I* > 2σ(*I*)
Monochromator: graphite	*R*_int_ = 0.013
*T* = 291(2) K	θ_max_ = 25.5º
φ and ω scans	θ_min_ = 2.4º
Absorption correction: multi-scan(SADABS; Sheldrick, 1996)	*h* = −10→10
*T*_min_ = 0.835, *T*_max_ = 0.910	*k* = −12→12
7557 measured reflections	*l* = −14→14

### Refinement

**Table:**

Refinement on *F*^2^	Secondary atom site location: difference Fourier map
Least-squares matrix: full	Hydrogen site location: inferred from neighbouring sites
*R*[*F*^2^ > 2σ(*F*^2^)] = 0.036	H-atom parameters constrained
*wR*(*F*^2^) = 0.100	*w* = 1/[σ^2^(*F*_o_^2^) + (0.0465*P*)^2^ + 0.2265*P*] where *P* = (*F*_o_^2^ + 2*F*_c_^2^)/3
*S* = 1.03	(Δ/σ)_max_ = 0.011
3741 reflections	Δρ_max_ = 0.15 e Å^−^^3^
326 parameters	Δρ_min_ = −0.17 e Å^−^^3^
66 restraints	Extinction correction: none
Primary atom site location: structure-invariant direct methods	

### Special details

**Table:**

Geometry. All esds (except the esd in the dihedral angle between two l.s. planes) are estimated using the full covariance matrix. The cell esds are taken into account individually in the estimation of esds in distances, angles and torsion angles; correlations between esds in cell parameters are only used when they are defined by crystal symmetry. An approximate (isotropic) treatment of cell esds is used for estimating esds involving l.s. planes.
Refinement. Refinement of F^2^ against ALL reflections. The weighted R-factor wR and goodness of fit S are based on F^2^, conventional R-factors R are based on F, with F set to zero for negative F^2^. The threshold expression of F^2^ > 2sigma(F^2^) is used only for calculating R-factors(gt) etc. and is not relevant to the choice of reflections for refinement. R-factors based on F^2^ are statistically about twice as large as those based on F, and R- factors based on ALL data will be even larger.

### Fractional atomic coordinates and isotropic or equivalent isotropic displacement parameters (Å^2^)

**Table:**

	*x*	*y*	*z*	*U*_iso_*/*U*_eq_	Occ. (<1)
Cl1	1.14956 (8)	0.27691 (8)	0.88868 (6)	0.0850 (2)	
S1	1.13849 (8)	0.12027 (6)	0.49405 (5)	0.06425 (19)	
C1	1.0219 (3)	0.4151 (2)	0.84413 (19)	0.0613 (6)	
C2	1.0746 (4)	0.5329 (3)	0.8166 (2)	0.0871 (9)	
H2	1.1790	0.5350	0.8185	0.105*	
C3	0.9723 (5)	0.6447 (3)	0.7868 (3)	0.0994 (11)	
H3	1.0086	0.7222	0.7673	0.119*	
C4	0.8182 (5)	0.6444 (3)	0.7854 (3)	0.0918 (9)	
H4	0.7492	0.7217	0.7667	0.110*	
C5	0.7651 (3)	0.5291 (2)	0.8119 (2)	0.0710 (6)	
H5	0.6596	0.5295	0.8113	0.085*	
C6	0.8666 (3)	0.4114 (2)	0.83974 (17)	0.0538 (5)	
C7	0.8062 (2)	0.28882 (19)	0.86731 (17)	0.0508 (5)	
C11	0.8598 (2)	0.17209 (19)	0.81392 (17)	0.0491 (5)	
C12	0.9819 (3)	0.13402 (19)	0.70679 (17)	0.0497 (5)	
C13	0.9865 (3)	0.2030 (2)	0.60054 (17)	0.0543 (5)	
C14	1.1993 (3)	−0.0064 (2)	0.59067 (18)	0.0556 (5)	
C15	1.1036 (3)	0.0151 (2)	0.69936 (18)	0.0535 (5)	
H15	1.1161	−0.0426	0.7635	0.064*	
C16	0.8792 (3)	0.3280 (2)	0.5679 (2)	0.0715 (7)	
H16A	0.7735	0.3348	0.6155	0.107*	
H16B	0.8756	0.3272	0.4863	0.107*	
H16C	0.9196	0.4022	0.5810	0.107*	
C17	1.3338 (3)	−0.1188 (2)	0.5501 (2)	0.0620 (6)	
C18	1.4225 (4)	−0.1226 (3)	0.4357 (3)	0.0872 (8)	
H18	1.4001	−0.0518	0.3835	0.105*	
C19	1.5454 (4)	−0.2320 (4)	0.3981 (3)	0.1079 (11)	
H19	1.6042	−0.2332	0.3209	0.130*	
C20	1.5801 (4)	−0.3361 (4)	0.4721 (4)	0.1062 (11)	
H20	1.6610	−0.4093	0.4457	0.127*	
C21	1.4962 (4)	−0.3333 (3)	0.5850 (3)	0.1009 (10)	
H21	1.5212	−0.4042	0.6366	0.121*	
C22	1.3741 (3)	−0.2260 (3)	0.6245 (3)	0.0823 (8)	
H22	1.3180	−0.2258	0.7023	0.099*	
C8	0.6691 (4)	0.2848 (3)	0.9689 (2)	0.0766 (7)	
F81	0.5227 (6)	0.3659 (5)	0.9349 (4)	0.0822 (12)	0.60
F82	0.6655 (6)	0.3429 (5)	1.0655 (3)	0.0934 (13)	0.60
F81A	0.5616 (11)	0.3714 (7)	0.9937 (8)	0.129 (4)	0.40
F82A	0.7574 (8)	0.2683 (6)	1.0735 (4)	0.104 (2)	0.40
C9	0.6459 (3)	0.1441 (2)	0.9782 (2)	0.0668 (6)	
F91	0.6251 (6)	0.0807 (5)	1.0781 (4)	0.1038 (17)	0.60
F92	0.5129 (5)	0.1513 (4)	0.9324 (4)	0.1069 (12)	0.60
F91A	0.7063 (9)	0.1016 (7)	1.0795 (5)	0.101 (2)	0.40
F92A	0.4934 (7)	0.1351 (6)	1.0116 (6)	0.098 (2)	0.40
C10	0.7729 (3)	0.0714 (2)	0.8807 (2)	0.0700 (7)	
F101	0.7425 (5)	−0.0114 (4)	0.8142 (4)	0.0895 (11)	0.60
F102	0.8871 (6)	−0.0155 (4)	0.9402 (5)	0.0906 (13)	0.60
F11A	0.6530 (8)	0.0595 (7)	0.8101 (5)	0.1013 (18)	0.40
F12A	0.8365 (11)	−0.0405 (6)	0.9036 (7)	0.105 (3)	0.40

### Atomic displacement parameters (Å^2^)

**Table:**

	*U*^11^	*U*^22^	*U*^33^	*U*^12^	*U*^13^	*U*^23^
Cl1	0.0693 (4)	0.1060 (5)	0.0829 (5)	−0.0199 (4)	−0.0218 (3)	−0.0053 (4)
S1	0.0813 (4)	0.0650 (4)	0.0454 (3)	−0.0197 (3)	−0.0090 (3)	0.0011 (2)
C1	0.0701 (14)	0.0671 (14)	0.0502 (12)	−0.0294 (11)	−0.0016 (10)	−0.0122 (10)
C2	0.100 (2)	0.095 (2)	0.0793 (18)	−0.0620 (18)	0.0027 (15)	−0.0207 (15)
C3	0.147 (3)	0.0639 (18)	0.091 (2)	−0.058 (2)	0.009 (2)	−0.0118 (15)
C4	0.129 (3)	0.0499 (14)	0.088 (2)	−0.0247 (16)	−0.0012 (18)	−0.0013 (13)
C5	0.0841 (17)	0.0511 (13)	0.0742 (15)	−0.0175 (12)	−0.0073 (13)	−0.0013 (11)
C6	0.0685 (13)	0.0472 (11)	0.0465 (11)	−0.0224 (10)	−0.0023 (9)	−0.0061 (8)
C7	0.0599 (12)	0.0462 (11)	0.0484 (11)	−0.0188 (9)	−0.0088 (9)	−0.0016 (8)
C11	0.0628 (12)	0.0438 (10)	0.0450 (10)	−0.0169 (9)	−0.0160 (9)	0.0020 (8)
C12	0.0646 (12)	0.0444 (10)	0.0448 (10)	−0.0184 (9)	−0.0148 (9)	−0.0015 (8)
C13	0.0725 (14)	0.0486 (11)	0.0466 (11)	−0.0207 (10)	−0.0160 (10)	0.0007 (9)
C14	0.0656 (13)	0.0533 (12)	0.0525 (12)	−0.0199 (10)	−0.0143 (10)	−0.0051 (9)
C15	0.0675 (13)	0.0471 (11)	0.0485 (11)	−0.0152 (9)	−0.0161 (10)	0.0002 (8)
C16	0.0987 (19)	0.0595 (13)	0.0574 (13)	−0.0132 (13)	−0.0271 (13)	0.0076 (10)
C17	0.0607 (13)	0.0640 (14)	0.0649 (14)	−0.0175 (11)	−0.0122 (11)	−0.0163 (11)
C18	0.0876 (19)	0.0861 (19)	0.0776 (18)	−0.0161 (15)	0.0043 (15)	−0.0154 (14)
C19	0.086 (2)	0.120 (3)	0.103 (2)	−0.013 (2)	0.0134 (18)	−0.040 (2)
C20	0.081 (2)	0.097 (2)	0.135 (3)	0.0066 (18)	−0.025 (2)	−0.041 (2)
C21	0.099 (2)	0.088 (2)	0.108 (3)	0.0151 (17)	−0.038 (2)	−0.0191 (18)
C22	0.0875 (18)	0.0765 (17)	0.0766 (17)	0.0023 (14)	−0.0240 (14)	−0.0102 (14)
C8	0.0927 (19)	0.0690 (16)	0.0682 (16)	−0.0414 (15)	0.0130 (14)	−0.0194 (13)
F81	0.0592 (15)	0.061 (2)	0.114 (4)	−0.0057 (13)	−0.002 (2)	−0.001 (2)
F82	0.111 (3)	0.117 (3)	0.0605 (18)	−0.061 (3)	0.013 (2)	−0.035 (2)
F81A	0.133 (9)	0.061 (3)	0.146 (9)	−0.027 (5)	0.081 (6)	−0.034 (6)
F82A	0.156 (6)	0.113 (4)	0.055 (2)	−0.086 (4)	0.019 (3)	−0.024 (3)
C9	0.0682 (15)	0.0615 (14)	0.0710 (15)	−0.0266 (12)	−0.0060 (12)	0.0075 (11)
F91	0.141 (4)	0.087 (2)	0.074 (2)	−0.055 (3)	0.025 (3)	0.0028 (16)
F92	0.070 (2)	0.098 (2)	0.159 (4)	−0.0215 (17)	−0.029 (3)	−0.014 (3)
F91A	0.165 (7)	0.084 (4)	0.045 (2)	−0.011 (4)	−0.028 (4)	0.014 (2)
F92A	0.076 (3)	0.071 (3)	0.140 (5)	−0.039 (2)	0.017 (4)	−0.007 (4)
C10	0.106 (2)	0.0546 (14)	0.0540 (13)	−0.0381 (13)	−0.0062 (12)	−0.0007 (10)
F101	0.131 (3)	0.069 (2)	0.081 (2)	−0.0582 (19)	−0.003 (2)	−0.0177 (19)
F102	0.102 (3)	0.063 (3)	0.089 (3)	−0.009 (2)	−0.0082 (19)	0.033 (2)
F11A	0.138 (5)	0.130 (5)	0.067 (3)	−0.094 (4)	−0.015 (3)	−0.013 (3)
F12A	0.141 (7)	0.033 (2)	0.109 (7)	−0.022 (3)	0.041 (5)	−0.003 (3)

### Geometric parameters (Å, °)

**Table:**

Cl1—C1	1.727 (3)	C16—H16C	0.9600
S1—C13	1.719 (2)	C17—C18	1.383 (4)
S1—C14	1.729 (2)	C17—C22	1.384 (4)
C1—C6	1.390 (3)	C18—C19	1.396 (4)
C1—C2	1.400 (3)	C18—H18	0.9300
C2—C3	1.366 (5)	C19—C20	1.350 (5)
C2—H2	0.9300	C19—H19	0.9300
C3—C4	1.361 (5)	C20—C21	1.356 (5)
C3—H3	0.9300	C20—H20	0.9300
C4—C5	1.378 (4)	C21—C22	1.382 (4)
C4—H4	0.9300	C21—H21	0.9300
C5—C6	1.398 (3)	C22—H22	0.9300
C5—H5	0.9300	C8—F81A	1.144 (8)
C6—C7	1.481 (3)	C8—F82	1.313 (4)
C7—C11	1.345 (3)	C8—F81	1.481 (6)
C7—C8	1.493 (3)	C8—C9	1.520 (3)
C11—C12	1.465 (3)	C8—F82A	1.566 (7)
C11—C10	1.506 (3)	C9—F91	1.288 (5)
C12—C13	1.377 (3)	C9—F92A	1.334 (6)
C12—C15	1.427 (3)	C9—F92	1.378 (4)
C13—C16	1.494 (3)	C9—F91A	1.400 (6)
C14—C15	1.357 (3)	C9—C10	1.511 (3)
C14—C17	1.477 (3)	C10—F12A	1.205 (7)
C15—H15	0.9300	C10—F101	1.315 (4)
C16—H16A	0.9600	C10—F102	1.446 (6)
C16—H16B	0.9600	C10—F11A	1.510 (6)
			
C13—S1—C14	93.38 (10)	C19—C20—H20	120.3
C6—C1—C2	120.2 (3)	C21—C20—H20	120.3
C6—C1—Cl1	120.61 (17)	C20—C21—C22	120.7 (3)
C2—C1—Cl1	119.2 (2)	C20—C21—H21	119.7
C3—C2—C1	119.8 (3)	C22—C21—H21	119.7
C3—C2—H2	120.1	C21—C22—C17	121.2 (3)
C1—C2—H2	120.1	C21—C22—H22	119.4
C4—C3—C2	121.1 (3)	C17—C22—H22	119.4
C4—C3—H3	119.5	F81A—C8—F82	65.8 (5)
C2—C3—H3	119.5	F81A—C8—F81	34.6 (5)
C3—C4—C5	119.6 (3)	F82—C8—F81	100.4 (4)
C3—C4—H4	120.2	F81A—C8—C7	124.0 (5)
C5—C4—H4	120.2	F82—C8—C7	117.8 (3)
C4—C5—C6	121.4 (3)	F81—C8—C7	107.7 (3)
C4—C5—H5	119.3	F81A—C8—C9	120.2 (5)
C6—C5—H5	119.3	F82—C8—C9	118.8 (3)
C1—C6—C5	117.9 (2)	F81—C8—C9	104.6 (3)
C1—C6—C7	122.0 (2)	C7—C8—C9	106.1 (2)
C5—C6—C7	120.1 (2)	F81A—C8—F82A	104.8 (6)
C11—C7—C6	129.06 (19)	F82—C8—F82A	39.3 (2)
C11—C7—C8	111.42 (18)	F81—C8—F82A	139.3 (4)
C6—C7—C8	119.51 (18)	C7—C8—F82A	100.1 (3)
C7—C11—C12	130.30 (18)	C9—C8—F82A	95.4 (3)
C7—C11—C10	110.21 (19)	F91—C9—F92A	70.4 (4)
C12—C11—C10	119.49 (17)	F91—C9—F92	107.3 (3)
C13—C12—C15	112.38 (19)	F92A—C9—F92	39.2 (3)
C13—C12—C11	124.34 (19)	F91—C9—F91A	34.3 (3)
C15—C12—C11	123.14 (17)	F92A—C9—F91A	103.0 (5)
C12—C13—C16	130.1 (2)	F92—C9—F91A	141.3 (4)
C12—C13—S1	110.26 (16)	F91—C9—C10	115.5 (3)
C16—C13—S1	119.55 (16)	F92A—C9—C10	127.6 (4)
C15—C14—C17	129.2 (2)	F92—C9—C10	99.4 (3)
C15—C14—S1	109.74 (17)	F91A—C9—C10	103.5 (4)
C17—C14—S1	121.03 (17)	F91—C9—C8	121.5 (3)
C14—C15—C12	114.24 (19)	F92A—C9—C8	114.0 (3)
C14—C15—H15	122.9	F92—C9—C8	104.8 (3)
C12—C15—H15	122.9	F91A—C9—C8	98.6 (3)
C13—C16—H16A	109.5	C10—C9—C8	105.60 (19)
C13—C16—H16B	109.5	F12A—C10—F101	69.7 (4)
H16A—C16—H16B	109.5	F12A—C10—F102	32.6 (4)
C13—C16—H16C	109.5	F101—C10—F102	101.7 (3)
H16A—C16—H16C	109.5	F12A—C10—C11	124.5 (5)
H16B—C16—H16C	109.5	F101—C10—C11	114.7 (2)
C18—C17—C22	117.3 (2)	F102—C10—C11	105.3 (3)
C18—C17—C14	121.4 (2)	F12A—C10—F11A	105.4 (5)
C22—C17—C14	121.3 (2)	F101—C10—F11A	38.6 (2)
C17—C18—C19	120.5 (3)	F102—C10—F11A	138.0 (3)
C17—C18—H18	119.8	C11—C10—F11A	105.3 (3)
C19—C18—H18	119.8	F12A—C10—C9	116.6 (4)
C20—C19—C18	120.9 (3)	F101—C10—C9	122.1 (3)
C20—C19—H19	119.6	F102—C10—C9	104.9 (3)
C18—C19—H19	119.6	C11—C10—C9	106.42 (18)
C19—C20—C21	119.5 (3)	F11A—C10—C9	93.3 (3)
